# Comparison of intraepidermal nerve fibre density among different age groups in healthy dogs

**DOI:** 10.1007/s11259-026-11196-2

**Published:** 2026-04-09

**Authors:** Mandara MT, G Giglia, S Arcaro, W Bergmann, R Zelli, A Di Salvo, G della Rocca

**Affiliations:** 1 Department of Veterinary Medicine, University of Perugia, Perugia, Italy; 2 Research Centre on Animal Pain (CeRiDA), University of Perugia, Perugia, Italy; 3 Division of Pathology, Faculty of Veterinary Medicine, Utrecht University, Utrecht, The Netherlands; 4 Department of Veterinary Medicine, Laboratory of Neuropathology, University of Perugia (ITALY), Via S. Costanzo 4, Perugia, 06126 Italy

**Keywords:** Dog, Intraepidermal nerve fiber, Pain perception, Skin biopsy

## Abstract

The intraepidermal nerve fiber (IENF) density is a key point in assessing pain perception in mammals. Its value tends to be different when addressed to different classes of age and different anatomical compartments. This study investigated the physiological range of IENF density within the hind limb in healthy dogs of different ages, in order to be aware of cutaneous pain perception expected in clinical practice and trace a starting point for the potential use of this method in the diagnosis of peripheral neuropathies. FFPE 8 mm punches on the hind limb were obtained from fifteen dogs equally divided into three classes of age: (1) newborns, (2) adults, (3) old dogs. The identification of intraepidermal nerve fibers was carried out by indirect immunofluorescence (IIF) and immunohistochemistry (IHC), using PGP 9.5 as a marker. The newborn dogs showed consistently higher IENF density compared to adult and old dogs, statistically significant when referred to the hind limb proximal site. A consistent gradient in IENF density between the proximal and the distal site of the hind limb was also observed in all classes of age. This study highlights the higher number of IENF in newborns compared with adults and old dogs. For this reason, pain perception in newborn dogs should be reconsidered in healthcare and medical procedures.

## Introduction

General somatic afferent pathways are involved in pain perception and about one half of the afferent axons in cutaneous nerves originate from nociception (Kennedy and Wendelschafer-Crabb 1993; Beiswenger et al. 2008; De Lahunta et al. 2009). These receptors are stimulated by mechanical, thermal and, for some extent, chemical stimuli. The nerve fibers arising from these cutaneous receptors are small and falling into the class of A-delta type (myelinated) or C-type (unmyelinated) fibers (Hudspith 2016; Glatte et al. 2019). These fibers terminate in layers I and V, and in layer II of the substantia gelatinosa in the dorsal horn of the spinal cord, respectively (Rees et al. 2010). For decades, human health care providers have believed that newborn infants were characterized by a weak or even absent ability to feel pain due to the immaturity of their nervous system (Puchalski and Hummel 2002). This conviction has long supported neonatal health care providers to perform surgical and other invasive procedures without analgesia or anaesthesia to infants (Puchalski and Hummel 2002). Further studies led to the current knowledge recognising that infants not only experience pain, but they experience it more acutely than adults (Reynolds and Fitzgerald 1995; Porter et al. 1999).

Moreover, pre-clinical studies evidenced that, in contrast to preterm infants, full-term infants develop pain memory resulting in an increased response to painful procedures in childhood and even in adulthood when exposed to short-term pain early in life (Puchalski and Hummel 2002). Such a similar misconception has also prevailed in the veterinary clinical practice. Albeit a number of studies in rats and sheep defined gestation and postnatal phases and times for the development of the sensory system (Rees et al. 2010), data about the distribution of peripheral sensory nerve fibers in different age classes are not available in animals. Besides that and regardless of pain sensitivity, it is interesting to note that to assess the density of intraepidermal nerve fibers (IENF) is a useful tool in the diagnosis of peripheral nervous system diseases and chronic metabolic disorders in humans (Kennedy et al. 1996, 1999; Polydefkis et al. 2004; Nolano et al. 2020). This is the case of length-dependent neuropathies and diabetic neuropathy, respectively, where IENF density assessment can also support the progression of disease or the effectiveness of treatments (Lauria et al. 2001; Beiswenger et al. 2008). Although in the recent years efforts to identify IENFs in dogs by immunofluorescence (IF) or immunohistochemistry (IHC) have been taken (de Medeiros et al. 2009; Laprais et al. 2017; Mandara et al. 2025), contrarily to humans, ranges of intraepidermal nerve fibre densities are still lacking in healthy dogs, nor have these densities been investigated in the diagnosis of peripheral small fibers affections. The aim of this study is to investigate the potential physiological range of the intraepidermal nerve fiber density within the hind limb in dogs of different ages, in order to gather insights on the cutaneous pain perception expected in clinical practice and to fix a starting point for the potential use of this method in the diagnosis of peripheral neuropathies.

## Materials and methods

### Sample selection

The dogs included in the study were divided into three different classes of age: (1) newborns (0–30 days old), (2) adults (4–9 years old), (3) old dogs (more than 10 years old). Each group consisted of 5 subjects, for a total of 15 dogs. Simple size calculation was not performed for this pilot study.

All dogs, spontaneously dead or submitted to euthanasia for reasons unrelated to the study, were referred for autopsy with owner’s consent. Dogs were not affected by peripheral neuropathies and metabolic diseases, nor by skin diseases. For each subject, two 8 mm skin punch biopsies were taken from the proximal (above the knee) and distal (tarsus) hindlimb, as these are two of the main anatomic reference sites used in cutaneous pain perception studies (Rees et al. 2010; Lauria et al. 1999) and in the diagnosis of peripheral neuropathies (Lauria et al. 1999, 2005, 2010; Shelton 2010; Nolano et al. 2020). Samples were obtained no more than 12 h after death, with the dogs stored in the refrigerator (+ 4 °C) pending sampling. Skin punch biopsies were immediately fixed in 10% neutral-buffered formalin for 4 days and subsequently paraffin-embedded following the protocol previously developed (Mandara et al. 2025).

## Identification of IENFs

The identification of intraepidermal nerve fibers was carried out by indirect immunofluorescence (IIF) and immunohistochemistry (IHC), using PGP 9.5 (ubiquitin C-terminal hydrolase) as a marker.

Following the European Federation of Neurological Societies (EFNS) guidelines (Lauria et al. 2005), three non-consecutive sections (each section selected every ten removed) were set up for each biopsy, placed on a slide with double polylysine coating.

Immunohistochemistry was performed on 5 μm thickness sections, with a rabbit polyclonal antibody anti-human PGP9.5 (1:450; GenTex, Milan, IT). After deparaffinization and rehydration, antigen retrieval was performed using a microwave for 20 min in Tris-EDA buffer solution (10mmol/L Tris base, 1 mmol/L EDTA, Ph 9.0). Endogenous peroxidase was blocked using 3% hydrogen peroxide in water for 5 min at room temperature. Afterward, the slides were covered with the primary antibody for 1 h in a humidified chamber at room temperature. Immunoreactivity was revealed by the avidin-biotin method using aminoethyl-carbazole substrate (AEC Substrate kit, abcam, Milan). Carazzi’s haematoxylin was used as the counterstain.

Indirect immunofluorescence was performed on 10 μm thickness sections, using a previously developed protocol (Mandara et al. 2025). Shortly, after deparaffinization and rehydration antigen retrieval was performed by thermic shock for 2 min at high temperature and 20 min at low temperature in Tris-EDTA buffer solution (10mmol/L Tris Base, 1 mmol/L EDTA, ph 9.0). Primary antibody anti-PGP9.5 (1:450; GenTex, Milan, IT) was incubated at 4 °C for 2 h in a humid chamber. Red fluorochrome (1:200; Goat pAb anti-Rabbit Alexa Fluor^®^ 594; abcam, Milan) was used as secondary antibody for 1.5 h at room temperature. Finally, the specimens were mounted with DAPI (blue fluorochrome; abcam, Milan, IT) and covered with a coverslip.

## Count of IENFs

The dermal-epidermal junction was used as a reference point to determine the length of each examined section in millimetres. Digital pictures were acquired using the Nis-Elements BR software (3.22.15 version) for both, IIF and IHC. For IIF, images of consecutive segments were taken for each section at x40 magnification, whereas for IHC, images covering the entire section were taken at x10. The acquired images were then analyzed using ImageJ software for length measurements.

At IIF, counting of intraepidermal nerve fibers (IENF density) was performed by fluorescence microscope (Olympus BX51, Olympus U-RL-T fluorescent lamp). The fiber count was carried out from images acquired at x40 magnification of consecutive segments along the entire length of the section. Both the fiber count and the length of the dermo-epidermal junction were determined using ImageJ software. Counts and measurements were repeated 3 times for each of the three non-consecutive sections, with at least one week in between. The IENF median value of all three measurements per section and the median value of all three sections for each biopsy were acquired for each dog.

At IHC, counting of intraepidermal nerve fibers was performed at x40 magnification (Mandara et al. 2025) using a bright field microscope (Olympus BX51). As for IIF, each of the three non-consecutive sections was read 3 times with at least one week in between as triplicate technical counts. The IENF median value for all three measurements per each section and the median value of all three sections for each biopsy were acquired for each dog.

In both IIF and IHC, only the fibers that crossed the dermo-epidermal junction were considered useful for counting (Lauria et al. 2005). For each punch biopsy, the final IENF density was referred to as the ratio of mean intraepidermal nerve fiber density value to the length of the section expressed in millimetres (number of fibers/mm). For each class of age (newborn, adult, and old dogs), the final IENF density value was again the median value among the 5 dogs belonging to the same age class.

Means and standard deviations were calculated for the IENF density on both IIF and IHC for each anatomical site. Statistical analysis was performed by the use of R/R-studio (Version 2024.09.1 + 394). Data were analysed at the patient level to avoid pseudo-replication. Age class was treated as a between-subject factor, while the anatomical site and staining technique (IHC or IIF) were treated as within-subject factors. To account for repeated measurements within the same subject, a linear mixed-effects model was fitted using restricted maximum likelihood. Age class, anatomical site, staining technique, and their interactions were included as fixed effects, and the patient number was included as a random intercept. Model assumptions were assessed by visual inspection of residual and quantile–quantile plots. Post-hoc pairwise comparisons were performed using estimated marginal means when appropriate, with p-values adjusted for multiple testing using the Holm method. Statistical significance was set at *p* < 0.05.

## Results

Fifteen dogs were considered in this study, including five dogs for each of the following classes of age: (1) newborns, 0–30 days; (2) adults, 4–9 years; (3) old dogs, more than 10 years. The first class (newborn ≤ 30 days) included three Australian Sheepdogs, a Jack Russel dog, and a large sized mixed breed dog. In the second class (from 4 to 9-year-old dogs) the five dogs were a Saint Bernard dog, a Springer Spaniel, an Italian hunting dog, a Cocker Spaniel, and an Akita Inu, respectively. In the third class (> 10-year-old dogs) the five dogs included a Maltese dog, a German Shepherd dog, a Jack Russel dog, a Labrador retriever dog, and a medium sized mixed breed dog, respectively. (Table 1).

**Table 1:** Intraepidermal Nerve Fiber density/mm through IIF and IHC referred to each animal and different hind limb site

AgeCategory	Breed	Age	Site of hind limb	IIF density(fibers/mm)	IHC density(fibers/mm)
Newborn (1–30 days)	Jack Russel	1 day	PP	7.30	0.51
			DP	9.98	5.22
	Australian shepherd	1 day	PP	9.21	4.15
			DP	2.70	1.49
	Australian shepherd	1 day	PP	6.35	2.87
			DP	7.70	2.13
	Australian shepherd	1 day	PP	12.11	5.76
			DP	2.60	0.88
	Mixed breed	3 days	PP	6.83	8.12
			DP	1.81	1.28
Adult (4–9 years)	Saint Bernard dog	4 years	PP	2.55	1.04
			DP	0.09	0.56
	Springer Spaniel	4 years	PP	0.08	0.57
			DP	1.26	0.88
	Italian hunting dog	8 years	PP	0.13	0.85
			DP	0.40	0.67
	Cocker Spaniel	9 years	PP	2.41	1.54
			DP	1.19	0.73
	Akita Inu	8 years	PP	0.85	0.00
			DP	0.08	0.00
Old(> 10 years)	Maltese	16 years	PP	2.97	1.25
			DP	1.27	0.80
	German Shepherd	11 years	PP	0.96	0.29
			DP	0.62	0.55
	Jack Russel	13 years	PP	0.87	1.09
			DP	2.31	2.87
	Mixed breed	11 years	PP	2.10	0.95
			DP	0.64	2.49
	Labrador retriever	10 years	PP	0.48	2.15
			DP	0.94	1.61

PP = proximal part; DP = distal part; IIF = indirect immunofluorescence; IHC = immunohistochemistry

**Fig. 1:**
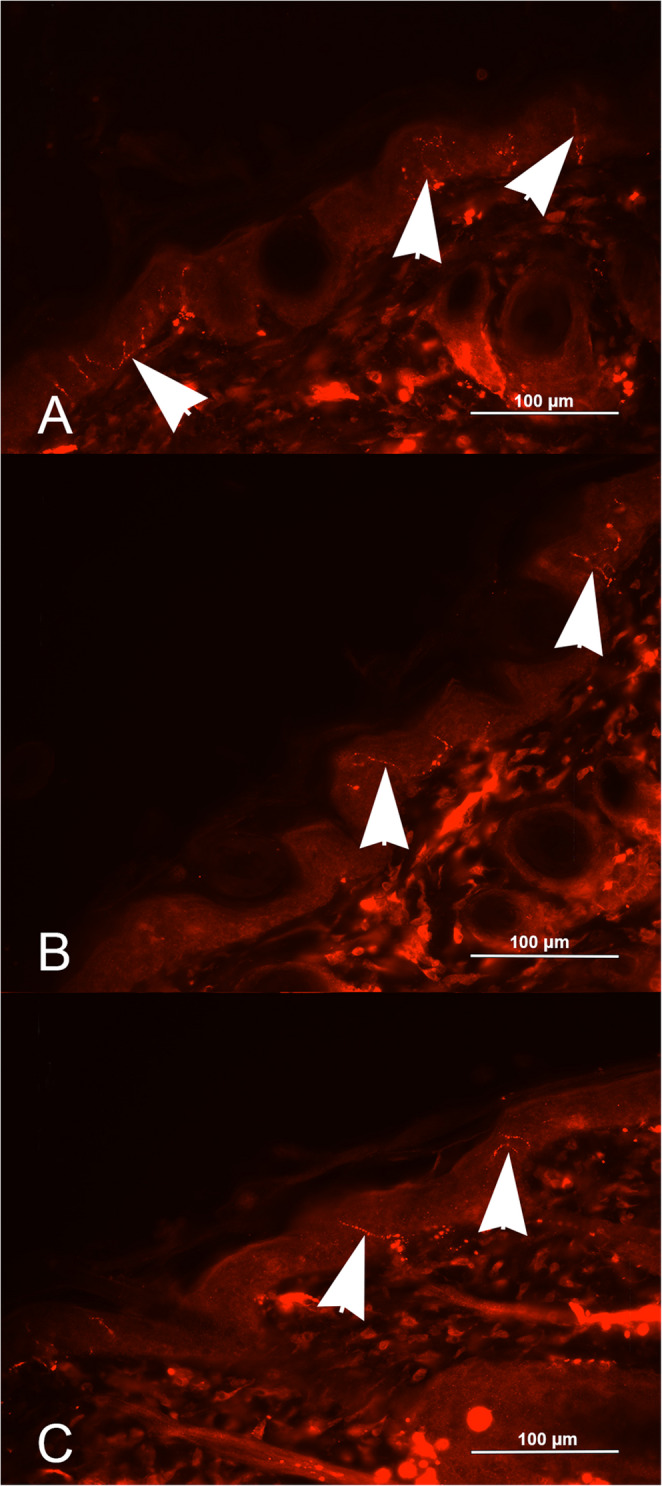
Skin biopsy. Proximal hind limb. Indirect immunofluorescence with evidence of intraepidermal nerve fibers (arrow heads). (**A**) Newborn class; (**B**) Adult dog class; (**C**) Old dog class. (400x; anti-human PGP9.5 rabbit polyclonal antibody)

At IIF, in the newborn dogs mean IENF density of 8.36 (sd = 2.36) and 4.96 (sd = 3.65) were defined in the proximal and distal hind limb, respectively. In the adult dog class, IENF mean density of 1.20 (sd = 1.20) and 0.60 (sd = 0.58) were defined in the proximal and distal hind limb, respectively. In the old dog class, IENF mean density of 1.47 (sd = 1.03) and 1.16 (sd = 0.69) were defined in proximal and distal hind limb, respectively (Table 2)(Fig. 1).

**Table 2:** Intraepidermal Nerve Fiber density/mm through IIF and IHC referred to each class of age and hind limb site

Age Category	Site of hind limb	IIF density (mean ± sd) (fibers/mm)	IHC density (mean ± sd) (fibers/mm)
Newborn (1–30 days)	PP	8.36 ± 2.36	4.28 ± 2.87
	DP	4.96 ± 3.65	2.20 ± 1.75
Adult (4–9 years)	PP	1.20 ± 1.20	0.80 ± 0.57
	DP	0.60 ± 0.58	0.57 ± 0.34
Old (> 10 years)	PP	1.47 ± 1.03	1.15 ± 0.67
	DP	1.16 ± 0.69	1.66 ± 1.01

PP = proximal part; DP = distal part; IIF = indirect immunofluorescence; IHC = immunohistochemistry

**Fig. 2:**
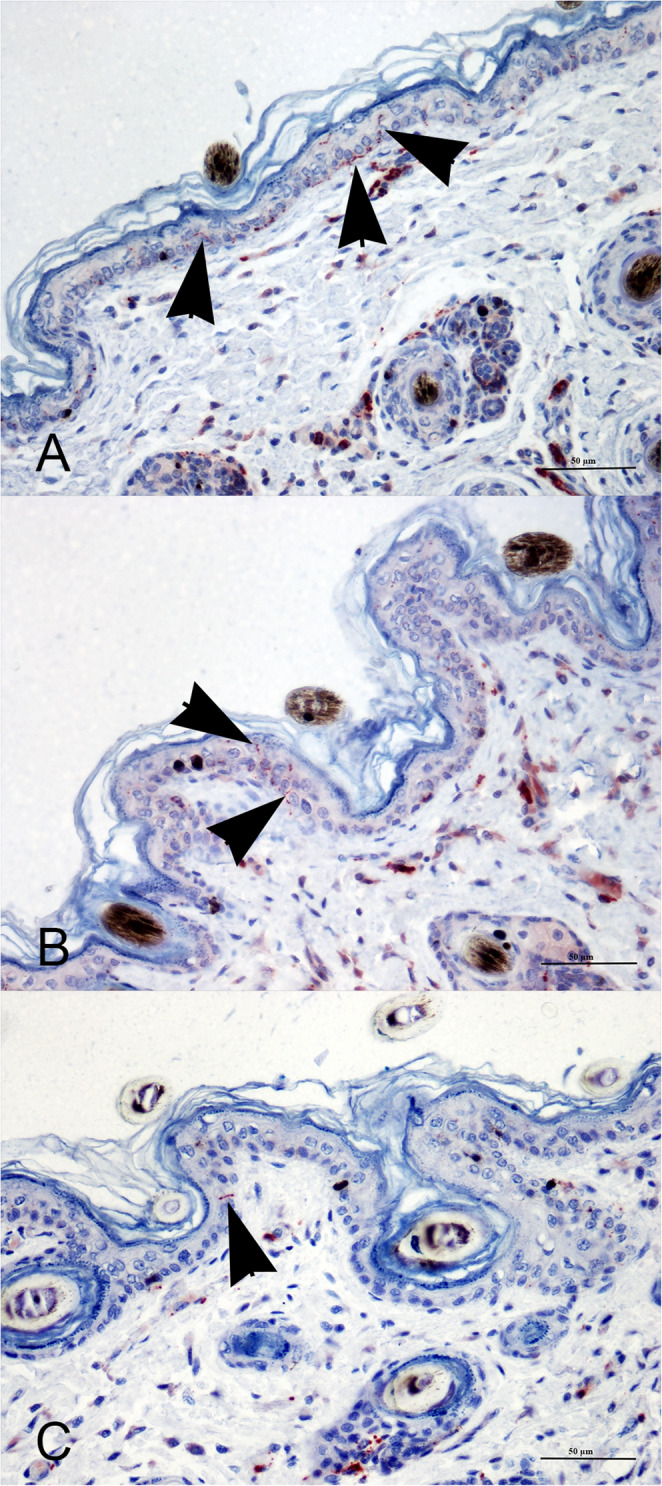
Skin biopsy. Proximal hind limb. Immunohistochemistry with evidence of intraepidermal nerve fibers (arrow heads). (**A**) Newborn class; (**B**) Adult dog class; (**C**) Old dog class. (400x; anti-human PGP9.5 rabbit polyclonal antibody)

At IHC, in the newborn dogs IENF mean density of 4.28 (sd = 2.87) and 2.20 (sd = 1.75) were defined in the proximal and distal hind limb, respectively. In the adult dogs, IENF mean density of 0.80 (sd = 0.57) and 0.57 (sd = 0.34) were defined in the proximal and distal hind limb, respectively. In the old dogs, IENF mean density of 1.15 (sd = 0.67) and 1.66 (sd = 1.01) were defined in the proximal and distal hind limb, respectively (Table 2) (Fig. 2).

## Statistical analyses

IENF density was analyzed using a linear mixed-effects model including class of age, anatomical site, and staining technique as fixed effects, and patient as a random effect. A significant main effect of the class of age was observed, with both adult and old dogs showing significantly lower IENF density compared to newborns (adults vs. newborns: estimate = − 3.48, *p* < 0.01; olds vs. newborns: estimate = − 3.14, *p* = 0.01). No significant difference was detected between adult and old dogs. Staining technique also had a significant effect, with IIF yielding higher IENF density values than IHC (estimate = + 4.08, *p* < 0.001). A trend toward lower IENF density at the distal site compared to the proximal site was observed, although this did not reach statistical significance (estimate = − 2.08, *p* > 0.05). A significant interaction between class of age and staining technique was detected, indicating that the difference between IIF and IHC varied across age classes. In particular, the IIF–IHC difference was more pronounced in newborns, and reduced in adult and old dogs (adults x IIF: *p* < 0.05; olds x IIF: *p* < 0.05). No significant three-way interaction among age class, anatomical site, and staining technique was observed. Post-hoc analyses with Holm correction confirmed that, at the proximal site, newborns exhibited significantly higher IENF density than both adult and old groups for both IHC and IIF staining. At the distal site, age-related differences were weak and did not reach statistical significance for IHC (*p* > 0.05). In contrast, IIF staining revealed significantly higher IENF density in newborns compared to adult (*p* < 0.01) and old dogs (*p* < 0.01), with no difference between adult and old groups (*p* > 0.05).

## Discussion

Acquiring knowledge on the nociceptive nerve fiber density expressed in both, healthy and pathological cutaneous tissue, is essential for the comprehension of potential peripheral pain perception in both, humans and mammals. This knowledge contributes on one hand in providing good management of health care and medical procedures (e.g., local anesthesia). On the other hand, investigating the preservation of the intraepidermal nerve fibers (IENFs) density might serve as a tool to understand the pathogenesis and clinical course of somatosensory length-dependent or metabolic peripheral neuropathies (Kennedy et al. 1996, 1999; Polydefkis et al. 2004; Nolano et al. 2020). In “dying-back” somatosensory neuropathies, IENF density values are significantly lower at the distal sites of the leg compared with healthy subjects, confirming the length-dependent loss of cutaneous innervation (Lauria et al. 2001). This is the case in diabetic neuropathy, in which a progressive decrease in IENF density from proximal to distal sites has been observed in skin biopsies obtained from multiple locations (Beiswenger et al. 2008), suggesting that small-fiber loss occurs in a length-dependent pattern. In addition, significant correlations between IENF density and other indices of diabetic neuropathy (warm and cold thermal threshold, heat pain, and pressure sensation) have been reported. Moreover, unlike sural nerve biopsy, skin biopsy allows re-evaluation and monitoring of disease progression, as well as assessment of treatment efficacy (Beiswenger et al. 2008).

It goes without saying that to explore all these conditions, there is a need for knowledge about the normal IENF density range in healthy animals, something that is still missing in the current literature for dogs.

In this study we defined the IENF density in 8 mm skin punches taken from the proximal and distal hindlimbs of healthy dogs of different ages. Tissue from the proximal and distal hindlimbs were taken as these are anatomic sites often investigated in the development of the somatosensory system (Rees et al. 2010) and the main anatomic reference sites in the diagnosis of peripheral neuropathies (Lauria et al. 1999, 2005; Shelton 2010). To process skin biopsy for this aim is not a routinary applied tool in clinical practice. In fact, to preserve the small IENFs, several fixation methods have been developed, unfortunately not routinely available, mainly favoring combinations of fixative solutions and freezing procedures prior to embedding (Lauria et al. 2010; De Madeiros et al. 2009). Moreover, the dense haircoat of canine skin can result in technical complications.

Supported by our previous results (Mandara et al. 2025), we investigated IENF density on formalin-fixed, paraffin-embedded (FFPE) skin punch biopsies, rather than on frozen samples, to compare immunofluorescence and immunohistochemistry protocols previously set up in our laboratory. However, consistently with the human EFNS guidelines (Lauria et al. 2005) we performed the IENF count/mm in three non-consecutive sections from the same skin biopsy. This method takes into consideration the patchy and clustering distribution pattern of nerve fibers in the epidermis (Lauria et al. 2005; de Medeiros et al. 2009) and as such avoids unsuspected bias in the final results. Moreover, the number of IENFs needs to be specifically referred to the thickness of sections adopted in the performed protocol. In fact, it is demonstrated that the value of IENFs tends to be higher in sections with a higher thickness (Mandara et al. 2025).

In this study, the newborn dogs showed consistently higher IENF density compared to adult and old dogs, although this difference resulted statistically significant only when referred to the hind limb proximal site and just above significance at the hind limb distal site. This result confirms the preliminary age-related trend we observed in the previous study (Mandara et al. 2025). However, the fibers number obtained in the two studies should be not compared since the final IENF densities have been evidenced and counted following different protocols. Indeed, the results of the previous publication (Mandara et al. 2025) maybe was wrongly influenced by the count in 5 consecutive x40 fields in a single section for each skin biopsy. By counting the IENFs in three non-consecutive sections, the results might show a much higher IENF density related to the opportunity to identify more IENF clusters due to their patchy distribution pattern (de Medeiros et al. 2009; Lauria et al. 2010).

Another interesting result obtained in this study is the apparent higher IENF density in the proximal hind limb (above the knee) compared to the distal site (tarsus). Considering the mean IENF density for each age group obtained by IIF, this gradient was almost double in newborns and adult dogs, whereas it tended to be lower in old dogs. However, statistical analysis did not confirm a significant difference. Moreover, although the different numerical scales of values obtained for IENF densities assessed by IIF and IHC methods, they did not result statistically different, except for the proximal site of hind limb in newborns. This result seems to support that both the techniques can be used interchangeably in this assessment. A similar proximal/distal hind limb gradient was less evident when IENF density was defined by IHC. A consistent gradient in IENF density between the proximal and the distal site of the hind limb is also well documented in humans, even when more than two sites were investigated in the leg (Lauria et al. 1999). In fact, in humans it occurs that the IENF density at the distal leg, calf and distal thigh is significantly lower than that at the proximal thigh and trunk (Lauria et al. 1999). As in dogs, a similar gradient of length-dependent epidermal innervation is present in different human age groups. Moreover, similarly to dogs, the linear IENF density does not consistently differ between adult (23–45 years) and old people (> 70 years) (Lauria et al. 1999). In humans, IENF density is lower among old adults (> 65 years), but not in middle-aged adults (45–65 years), compared to young (18–44 years) (Ekman et al. 2024). Moreover, no significant differences are reported between young (18–44 years) and middle-aged people (Ekman et al. 2024). However, although in humans IENF density is described to decrease from young to old adults, with a cut off decline of 0.54 fiber/mm for every 10 years (Provitera et al. 2016), newborn or infant age groups have not been considered in the evaluated dataset. In our study, a greater effect of the age on the IENF density was detected comparing newborn dogs with adult and old dogs, with the number of fibers up to more than five times higher in neonate dogs.

As for the linear density of IENF/mm, the differences between humans and dogs should be interpreted in light of the different species. However, different immunostaining protocols applied in different laboratories, especially referred to the thickness of sections, should also be considered. To this concern, most recently, IENF densities evaluated in standard 5 μm FFPE sections by IHC produced new normative data for potential diagnostic use in humans (Ekman et al. 2024), which seem much closer to those observed in the adult and elderly canine categories in our study. Although lower values of IENF density are to be expected, the use of standardized 5 μm FFPE sections is preferred for a more convenient handling in clinical pathology and diagnostic laboratories.

Along with the previous analysis (Mandara et al. 2025), we have sufficient elements to confirm that, based on peripheral intraepidermal nociceptive fibers, puppies within a month of age could express a higher pain perception compared to adult and old dogs. This result should be taken into great consideration in health care and medicine procedures applied to newborn dogs. As for old dogs > 10 years, we expected a more consistent decrease of linear IENF density compared to adult dogs. This apparent flattening of data could however be the result of multiple dogs in the adult and old dog groups with an age proximal to the cutoff. However, a similar continuity between the linear IENF density of the leg in adult and old people is also reported in healthy humans (Lauria et al. 1999). More recently, in humans it was found that the IENF density among old adults (> 65 years) is lower than in young (18–44 years), and no significant differences have been reported between young (18–44 years) and middle-aged adults (45–65 years) groups (Ekman et al. 2024).

What seems to be confirmed is that for the same antibody used, IIF is a more refined technique than IHC, as it is able to reveal a higher number of IENFs (Nolano et al. 2015). Nevertheless, the IENF counting protocol we used for IIF sections proved to be much more elaborate and considerably more time-consuming or in any case more expensive than IHC when it would be routinely applied for diagnostic purposes. In fact, to preserve the reaction, fiber counting was not performed directly on the sections, as for IHC, but for each section on consecutive images captured under the microscope. Of concurring opinion is a recent work aimed to redefine IENF density values in humans in 5 μm FFPE skin biopsies through IHC as a more applicable method in clinical practice (Ekman et al. 2024).

Considering the low number of animals per each class of age, one of the main limitations of this study consisted in the wide heterogeneity of canine breeds included in each of them. Perhaps the adult dog group escaped this limitation by being represented by four out of five animals being a medium-size breed. We are convinced that this parameter should be investigated more in depth and so not to fall into future bias. However, contrarily to the anatomic site, the body mass index which could be one of the main parameters influencing the differences in IENF density among different breeds, is reported not to have a significant role in humans (Provitera et al. 2016). For a more specific diagnostic purpose it would be great to reproduce this study in a large canine cohort with a more homogeneous population within groups. Additionally, to fully validate this method as a diagnostic tool, a similar study should be performed in canine breeds known to be predisposed to peripheral neuropathies, both healthy and diseased. At the same time, it would be worthwhile to investigate potential differences of IENF density between male and female dogs (Ekman et al. 2024). In humans, it has been reported that women have a density of 1.0 fiber/mm higher than men (Provitera et al. 2016). To learn more broadly about skin pain sensitivity in dogs and improve animal care during medical practice, it would also be of great interest to investigate IENF density in more anatomical sites than the hind limb and to correlate this data with functional assessments of the pain perception, such as behavioural pain responses or quantitative sensory testing.

In conclusion, both IIF and IHC confirmed that newborn dogs have a significantly higher IENF density compared to adult and old dogs, supporting the absolutely non negligible cutaneous pain perception in puppies. This result can no longer be neglected in veterinary practice and handlings applied to newborns. Moreover, both the IIF and IHC techniques are confirmed to be useful for counting IENF in routinely formalin fixed paraffin embedded skin punches. However, the IIF count protocol presents challenges on the routine analysis, and when using the IHC method, one must contend and take in account the lower IENFs density values. Regardless the technique applied in the laboratory, IENF density in FFPE skin biopsy punch could represent a potential new tool in evaluating changes of IENF density occurring in somatosensory and metabolic peripheral neuropathies in dogs. Future researches are required to further validate this tool on dogs affected by peripheral neuropathies.

## Data Availability

The data generated during and/or analysed during the current study are available from the corresponding author on reasonable request.
